# Understanding breast cancer risk factors: is there any mismatch between laywomen perceptions and expert opinions

**DOI:** 10.1186/s12885-022-09372-z

**Published:** 2022-03-23

**Authors:** E. Manouchehri, A. Taghipour, A. Ebadi, F. Homaei Shandiz, R. Latifnejad Roudsari

**Affiliations:** 1 Department of Midwifery, Faculty of Nursing and Midwifery, Mashhad Medical Sciences, Islamic Azad University, Mashhad, Iran; 2 Department of Midwifery, School of Nursing & Midwifery, Mashhad University of Medical Sciences, Mashhad, Iran; 3 Present Address: Social Determinants of Health Research Center, Mashhad University of Medical Sciences, Mashhad, Iran; 4 Department of Epidemiology, School of Public Health, Mashhad University of Medical Sciences, Mashhad, Iran; 5 Behavioral Sciences Research Center, Life style Institute, Baqiyatallah University of Medical Sciences, Tehran, IR Iran; 6 Nursing Faculty, Baqiyatallah University of Medical Sciences, Tehran, IR Iran; 7 Cancer Research Center, Mashhad University of Medical Sciences, Mashhad, Iran; 8 Nursing and Midwifery Care Research Center, Mashhad University of Medical Sciences, Mashhad, Iran

**Keywords:** Breast cancer, Qualitative research, Risk factor, Perception, Expert opinion

## Abstract

**Background:**

Women’s perception and knowledge of breast cancer signs, symptoms, and risk factors could be conducive to breast cancer risk management and interventions. The present study aimed to explore Iranian laywomen perceptions and expert opinions regarding breast cancer risk factors.

**Methods:**

This qualitative study was conducted from March to November 2019 in Mashhad, northeast of Iran. Through purposive sampling, 24 laywomen (women with and without BC) and 10 experts of different fields including oncology, surgery, gynecology and reproductive health were selected. Data collection was carried out using semi-structured interviews, which was mainly focused on the participants’ understanding and perception of BC risk factors. The data was analyzed utilizing conventional content analysis developed by Graneheim & Lundman. Components of trustworthiness, including credibility, dependability, confirmability, and transferability were considered.

**Results:**

The main category of risk factors, which emerged from the lay participants’ data analysis, were “unhealthy lifestyle and habits” , “hormonal influences”, “environmental exposures”, “Individual susceptibility “and “belief in supernatural powers”. The experts had similar perspectives for certain risk factors, yet not for all. The category of “Individual history of disease” was emerged only from experts’ interviews.

**Conclusion:**

In the present study, the lay participants’ perception concerning BC risk factors was found to be a mixture of cultural beliefs and the scientific knowledge dispersed by the media, internet, and health services. Primary prevention approaches, including awareness of breast cancer risk factors, are required for women to make improved health-related choices.

## Introduction

Breast cancer (BC) is a multifactorial disease whose development involves various factors [1]. Some of the factors that can affect its risk include heredity and genetics and the factors affecting the amount of endogenous hormones, such as reproductive factors, exogenous hormone intake, lifestyle, anthropometric characteristics, increased breast density in mammography, and benign breast diseases [2].

Risk perceptions are contextual evaluations of knowledge, which help people to understand their vulnerability and make health-related decisions [3]. Since realistic and accurate risk perception can contribute to appropriate health behaviors, enhancing public awareness on risk factors of diseases is believed to be one of the most important objectives of risk communication [4].

According to a study on Iranian women’s perception regarding control and prevention of BC, lack of awareness of BC risk factors is a major issue that affecting women’s BC screening behaviors [5]. Certain studies have reported misconceptions about the risk factors of BC, such as the use of antiperspirants or breast size as the cause of BC [6].

A qualitative study to explore BC risk perception in women with increased risk showed that the interpretation of BC risk factors by the participants was superimposed with their sense of social norms about how they could understand and control their BC risk; they felt as though they had to rationalize thoughts and concepts that were inconsistent with their behavior [7]. Another paper was conducted on Iranian immigrants to explore their beliefs and explanations about BC and its causes. It revealed a hybrid of both traditional and scientific beliefs about BC and its risk factors [8]. Additionally, a study on women with a family history of BC demonstrated a lack of information in this group concerning advancing age, early age at menarche, and late age at menopause as risk factors for BC [9].

Several quantitative articles have also examined the extent to which public is aware of the risk factors of various types of cancer. The results of these studies have indicated that participants had limited knowledge about BC risk factors, such as obesity, oral contraceptives, late menopause, and early onset of menses [10–13].

Patients perceive the risk of illness based on previous personal or family experiences with their disease or information they have received from health care providers or the mass media. Risk perception may be specific to individuals and the risk perceived by lay people may differ significantly from those of the experts [14]. Therefore people’s perception and how they perceive the risk of illnesses in different communities require further studies [14]. The results of a systematic review of 22 studies indicated that laywomen beliefs about the causes of cancer may not always be consistent with the judgment of experts. Specifying the range of mismatch is of great importance in order to provide prevention programs and help the promotion in understanding laywomen support necessities [15]. The experts’ sources of knowledge and information about health risks are acquired through university textbooks, scientific journals, symposiums, meetings, and conferences, as well as experiences of medical doctors through their clinical practices [16]. Meanwhile, laywomen risk perception is intuitive and is less scientific than experts [17].

Under any circumstances, providing information about a disease and its management to a target group is impossible without taking their beliefs and values about disease risk factors into consideration [8]. Qualitative studies highlighted that culture, experiences, knowledge, and beliefs can affect individuals’ perceptions and attitudes toward diseases [18, 19]. Thus, people’s perception and how they perceive the risk of an illnesses in different populations require further investigation via qualitative mode of inquiries. Accordingly, in the present study, the qualitative approach was adopted as risk perception is a complicated phenomenon and is affected by multiple factors. In order to investigate the perceptions of people and to provide an in-depth understanding of human behavior and demonstrate the variability within a given population, qualitative research was employed; it is known to be an appropriate method for providing a deeper understanding of individuals’ perceptions regarding BC risk factors [20].

Due to the insufficient studies on this subject using the qualitative approach in Iran, the current study was conducted to identify and compare perceived risk factors of BC among experts and laywomen in Iran.

## Materials and methods

A qualitative descriptive study was employed to explore Iranian women’s perceptions and experts’ opinions about the risk factors of BC. This method is suitable for research that questions human views. Based on this method, reality varies from person to person and qualitative description seeks to accurately describe a phenomenon like risk perception while not getting too far from its literal description interpreting the results [21].

The laywomen in our study were recruited through purposive sampling method in Mashhad, a metropolis in northeastern Iran. The lay participants were divided into two groups; the first group comprised women with BC referred to hospitals of Mashhad University of Medical Sciences or private clinics in Mashhad for chemotherapy. The second group consisted of women without BC or any signs or symptoms of BC, who were apparently healthy; they were patients’ relatives, who were recruited from hospitals or clinics or were healthy people recruited from other settings, like universities or workplaces.

To identify and recruit eligible experts, snowball sampling technique was applied, which is a convenient sampling method. In this method, the subjects of a study introduce future subjects among their peers [22]. For this purpose, specialists of different fields, such as oncology, surgery and reproductive health, were asked to participate in this study. The experts who met the following criteria were eligible: 1) currently participating in BC management; and 2) having at least 5 years of BC management experience.

For data collection, an open-ended semi-structured interview guide was developed based on a review of the literature and discussion with the research team. The interview schedule consisted of two groups of questions: the main interview questions and probing questions. The interviews were conducted by the first author (EM) with a background in health research and clinical gynecology. Questions were about participants’ understanding and perception of BC risk factors. The questions for women without BC were: “How much do you think you are at risk for BC?, Is there anything you think can increase or decrease your risk of BC?” . The questions for women with BC were: “What do you think are the conditions in your life that have caused the disease? Is there anything you think can increase or decrease the risk of BC?”. The questions for experts were: “Based on your experience, what are the risk factors for BC? What are the factors that increase the prevalence of BC in Iranian women?”. Probing questions such as “Can you explain more?” Or “What do you mean by this sentence?” were also used to provide further explanations.

The interviews were conducted face to face and continued until achievement of data saturation when no new information emerged from the interviews [22]. They were conducted and analyzed in Persian language. Subsequently, only the passages used as quotes were translated in English. It is noteworthy that the quotations from non-English interviews could be utilized if the researchers have further awareness of sensibility toward dealing with language and translation issues [23]. For this reason, quotations were translated by a bilingual expert who was fluent in both Persian and English languages. The interviews lasted for approximately 70 to 90 min for the laywomen and 30 to 45 min for the experts. Interviews were audio-recorded and then transcribed verbatim by a member of the research team (EM). The data were collected from March to November 2019.

The analysis was carried out using conventional qualitative content analysis developed by Graneheim and Lundman [24]. To this end, de-contextualization and re-contextualization steps were performed; the text was primarily read several times to get a general sense and insight. Afterwards, the text was broken into pieces and the meaning units were identified. The meaning units were then condensed and coded. In the re-contextualization step, the codes were sorted based on their similarities and differences and the subcategories and categories were generated. Transcripts of the initial three interviews were reviewed by the research team members (AT, AE, and RLR), with the objective of establishing a preliminary coding pattern that was used for subsequent analyses. All the transcripts were then analyzed by two researchers (EM and AT) and the interpretations was discussed with the third researcher (RLR) who was an experienced scholar in qualitative methodology and the supervisor of the research project.

Since data collection and analysis were complementary, the process was iterative, reflexive, and interactive; for example, the data collected during subsequent interviews were used to modify code labels or meanings found during earlier interviews. After the initial coding of the all transcripts, the team members discussed the coding strategy and the initial codes to reach a consensus and merge individual codes into subcategories and then the main categories [25]. Data analysis was supported by MAXQDA 12 software.

The four criteria presented by Lincoln and Guba, namely credibility, reliability, confirmability, and transferability, were examined in order to confirm the validity and reliability of this qualitative study [26]. In order to provide credibility, the researcher continued the interviews until the achievement of data saturation and selecting the informant participants. The researcher’s interest in the field of study, prolonged engagement and immersion in the data, and interviewing various subjects were among the factors that guaranteed credibility. To meet dependability, the possibility of doing an audit trial was provided through keeping the records of the raw data, transcripts, and a reflexive journal, which could help other researchers to systemize, share, and cross-reference data to make the study and its findings auditable. In addition, the present study was conducted as a team-work with the supervision of experts, which made both data credibility and dependability possible.

To ensure confirmability, a number of qualitative researchers were consulted and the researcher tried to describe the method of the current study with details. To increase the transferability, purposive sampling was used and interviews were conducted with different participants with the maximum diversity and direct quotations and examples were provided.

Prior to each interview, the participants were explained the objectives of the research, the reason of recording the interview, voluntary participation, data confidentiality, and the right to withdraw from the study at any time without any prejudice; informed consent was also obtained. All the information obtained from the participants was kept confidential and anonymous. That said, the subjects were given codes used in the study instead of their names. Gift cards (500,000 Rials, equivalent to $12) were provided to all of them as an appreciation for their participation. Ethics approval was granted by the Mashhad University of Medical Sciences, Mashhad, Iran under the code of IR.MUMS.NURSEREC.1397.034.

## Results

Tables 1 and 2 display the characteristics of the participants. 24 laywomen and 10 experts were interviewed. Following coding the interviews, 492 initial codes were generated and the codes related to the research objectives were finally divided into eight subcategories and five categories for the lay participants and 12 subcategories and five categories for the experts (Tables 3).

**Table 1:** Demographic profile of lay women (*n* = 24)

Lay participants characteristics	NO. (%)
**Age**	
20-29	2 (8)
30-39	9 (38)
40-49	5 (21)
50-59	7 (29)
60-69	1 (4)
**Literacy levels**	
Primary	5 (21)
Diploma/secondary	6 (25)
University	13 (54)
**Occupation**	
Housewife	12 (50)
Employed	12 (50)
**Marital status**	
Married	15 (64)
Widow	1 (4)
Divorced	4 (16)
Single	4 (16)

**Table 2:** Demographic profile of experts (*n* = 10)

Experts characteristics	NO. (%)
**Specialty**	
Oncology	3 (30)
Gynecology (oncology fellowship)	2 (20)
Surgery (general- breast)	2 (20)
Reproductive Health	1 (10)
Palliative Care	1 (10)
Nutrition	1 (10)
**Gender**	
Male	5 (50)
Female	5 (50)
**Years of BC management experience**	
10 – 20	3 (30)
≥20	7 (70)

**Table 3:** Subcategories and categories emerged from the laywomen and experts interviews

Category	Subcategory	Shared Perceived risks	Risks only perceived by laywomen	Risks only mentioned by Experts
Unhealthy lifestyle and habits	Unhealthy lifestyle behaviors	✓		
	Nutritional behaviors and habits	✓		
Hormonal influences	Reproductive history			✓
	Exposure to exogenous hormones	✓		
Environmental exposures	Exposure to magnetic fields	✓		
	Exposure to ionizing waves			✓
	Exposure to environmental pollution	✓		
History of disease	Previous breast problems			✓
	Health problems			✓
Individual susceptibility	Family history of cancer	✓		
	Genetic background	✓		
	Anthropometric characteristics			✓
	Demographic characteristics			✓
	Psychological factors		✓	
Belief in supernatural powers	Divine destiny and providence		✓	
	Karma effect		✓	

Laywomen and experts had similar perspectives for some risk factors, but not for all of them.

### Shared categories between laywomen and experts

There were several shared categories regarding risk factors of BC, including “unhealthy lifestyle and habits”, “hormonal influences”, “environmental exposures” and “Individual susceptibility “.

#### Unhealthy lifestyle and habits

There was consensus amongst participants (Laywomen and experts) that the individual lifestyle is likely to lead to the development of BC.

##### Unhealthy lifestyle behaviors

Almost all the participants were aware of a number of lifestyle-related risk factors whereas there was a lack of information about some other factors.

**Shared perceived risks:** Many lifestyle factors are likely to enhance the risk of developing BC. Some of the laywomen perceived the risk related to unhealthy lifestyle in agreement with the experts’ opinions. One of the lay participants said:

"I did not always like the habit of smoking of my husband. I used to say that a problem would finally happen for me or for our children, which did ... I knew that poor nutrition could lead to a variety of cancers but I did not know that being single and late marriage could also cause it (BC). I heard these things here … ". (P.5)

Similarly, one of the experts said:

"A factor that affects BC is inactivity. A sedentary lifestyle could be an effective factor even without obesity … ..Undoubtedly, diet, smoking, hookah, and passive smoking are among the risk factors, the person may not be a smoker or use hookah, but might be exposed to smoke in the family or workplace. Moreover, air pollution, unhealthy foods, full-fat diet, obesity, being overweight are the factors leading to BC. "(P.31)

Excessive and constant use of cosmetics was seen in both lay participants’ and experts’ opinions. One of the BC participants said:

"I am a person who always makes up. I believe there might be a relationship. Hair color and makeup … ."(P.12)

**The risks perceived only by the laywomen:** Some of the risk factors associated with an unhealthy lifestyle, which were perceived by the lay participants, were not mentioned by the experts like waxing. Some of the participants with BC, believed that BC could be contagious.

**The risks mentioned only by the experts:** Certain risk factors were only stated by the experts and none of the lay participants mentioned them, for instance, night-shift work and alcohol consumption.

##### Nutritional behaviors and habits

Regarding nutritional behaviors and habits, there were also similarities and differences concerning the perceived risks of both groups of the participants.

**Shared perceived risks:** The risk factors perceived and cited by both laywomen and experts included no use of fresh fruits and vegetables, use of processed, packed, and canned foods, and consumption of fast foods and food additives. One of the lay participants with a positive history of BC said:

"I have heard that chemical fertilizers are used indiscriminately in agriculture. Well, contaminated Fruits and meat and consumption of preservatives and fast foods are nowadays prevalent among people. These effects are not still clear; for example, ten years later, people might get BC while not knowing the reason . … ." (P.5)

An oncologist stated that:

“It is true that higher fat in the diet, vitamin D deficiency, omega 3 deficiency, and diets containing low fish and aquatic food could be risk factors for BC”. (P.25).

**The risks perceived only by the laywomen:** One of the participants diagnosed with BC said:

"I have seen that one of our relatives empties the water of kettle. She says boiling kettle water for several times is carcinogenic. She said it affects nerves and is carcinogen … .." (P.2)

A participant whose daughter was receiving chemotherapy stated:

"Palm oil is dangerous. I do not eat much meat at all. The doctor also told my daughter not to eat red meat now. Vegetables are better." (P.14)

**The risks mentioned only by the experts**: None of the participants were aware of high-calorie intake, Vitamin D deficiency, consumption of fatty foods and sauces, omega-3 deficiency, and changes in the diet of the community in general, as risk factors.

#### Hormonal influences

The perceived risk in this category was “Intake of exogenous hormones” and “individual’s reproductive history”.

##### Intake of exogenous hormones

All participants agreed on the use of exogenous hormones as a BC-related risk factor.

**Shared perceived risks:** The perceived risk of laywomen in this category was only the use of exogenous hormones, such as oral contraceptive pills and hormonal medications, which was in accordance with the experts’ opinions.

"One of the things I have heard is that I should not take hormonal pills...Even the contraceptive pills, as I heard, are not good at all." (P.16)

"Any use of exogenous hormones is important. Hormone replacement therapy is associated with a greater risk of BC. Once estrogen rises either endogenously or exogenously, a woman is more likely to develop BC..." (P.26)

##### Individual’s reproductive history

None of the laywomen were aware of reproductive history as a risk factor for BC.

**The risks mentioned only by the experts**: The factors related to the reproductive history were addressed only by the experts and all of them had a consensus in this regard. One surgeon said:

"Being nulligravid, having pregnancy after the age of 30 and limited parity, and pregnancy without breastfeeding could also be risk factors … .." (P.31)

#### Environmental exposure

Regarding environmental exposure, certain risk factors perceived by the laywomen were in line with experts’ opinion and some were different:

##### Exposure to magnetic fields

All participants took a variety of magnetic fields and environmental pollutants into account as BC risk factors.

**Shared perceived risks**: In this category, the laywomen perceived risks were related to exposure to electro-magnetic fields, such as telecommunication towers, modem, mobile phones, and Wi-Fi waves. A 30- years-old participant without BC stated:

"Large telecommunication towers noise. … . they have a terrible effect. I am sure it significantly affects our health … … At night, when I want to sleep, I charge my phone in another room." (P.16)

A housewife participant with BC stated: "I think these waves are very important. In some areas of Mashhad, there are much more waves. The telecommunication noise causes a lot of diseases, which (BC) has unfortunately increased so much in Iran … ." (P.11)

Nonetheless, the experts had different opinions in this regard although some experts had similar views with the laywomen. One of the oncologists with 28 years of experience said:

"It is unlikely that these waves could change DNA due to their long wavelength and low frequency. They cannot penetrate DNA. Theoretically, the waves from the electromagnetic spectrum can be carcinogenic if they are ionized. That means short wavelengths, high frequencies, and high-energies waves can damage DNA. "(P.26)

The most frequently mentioned risks about environmental pollution concerned water and air pollution.

##### Exposure to ionizing waves

**The risks mentioned only by the experts**: No perceived risks of exposure to ionizing waves was expressed by the laywomen. Meanwhile, the experts mentioned mammography or other diagnostic radiographies, chest radiography during puberty, chest radiotherapy for various causes, such as BC, and exposure to environmental ionizing radiation as BC risk factors:

"Ionizing waves, such as X-rays, and diagnostic imaging with X-ray, like CT and conventional radiographs, can all be carcinogenic." (P.26)

#### Individual susceptibility

Concerning individual susceptibility, there were similarities and differences between the laywomen and experts. The family and genetic background were mentioned in both groups. However, in the laywomen, “psychological factors” and in the group of experts, “anthropometric characteristics” and “demographic characteristics” were perceived.

##### Family and genetic background

There were differences and similarities between the perception of the participants concerning family and genetic background.

**Shared perceived risks:** The perceived risk factors by the laywomen were limited to family history of BC, ovarian cancer, hereditary background, and genetics. In this regard, one of the participants stated:

"We are 100% at risk. Because it is said that genetics plays a key role in the occurrence of this cancer and since my mother's BC was aggressive, we consulted several specialists; the first thing they said was that her daughters and sisters follow up the disease seriously...." (P.20)

**The risks mentioned only by the experts**: The experts pointed broader risk factors in this field, such as family history of colon and gastric cancer, paternal family history of BC, family history of glioma or multiple cancers in young relatives, the number of affected relatives, and history of BC in a male family member. One of the experts mentioned:

"The next issue is whether there was anyone in the family with cancer under the age of 40. Has anyone had a history of bilateral cancers? Did a man in their family have cancer? The presence of a father, brother, or close male relative who has had cancer is closely linked to the brca2 gene. After that, sarcoma, glioma, in other words, some brain tumors, in relatives under the age of 45 can increase the risk. One thing that is of particular importance is the high risk of BC if two or more people from the father's family of a woman have had cancers … " (P.29)

##### Psychological factors

Psychological-related factors were mainly perceived by the lay participants as BC risk factors.

**The risks perceived only by the laywomen:** One of the participants with a history of BC said:

"I think it could be more attributed to a nervous breakdown and not expressing problems. Those who are a bit introverted are more likely to get it (BC). I saw this in our family and my friends. BC is more prevalent in those who keep the problems to themselves compared with that in those who open up; for example, once they get upset and express their problem, they will get calm and everything will end. However, sensitive and emotional people are more at risk." (P.11)

Another participant in this regard said:

"I think it is just grief and a nervous shock at once. My father had a cardiac arrest four years ago. I was very dependent on him. He was fine and healthy. He came home from work and died suddenly. It was a huge shock to me ..." (P.10)

##### Anthropometric characteristics

**The risks mentioned only by the experts**: Anthropometric factors were only mentioned by the experts.

"An important factor in Asian and Iranian countries, and not in European and American populations, is that they have visceral or abdominal obesity. People with BC often have a specific phenotype that looks like a samovar. This means that their necks are shorter, their waist is bigger, and their hips are smaller … .." (P.28)

##### Demographic characteristics

**The risks mentioned only by the experts**: Some factors, like age and sex, were found in the experts’ interviews:

"Gender and age are also the most important risk factors (for BC). Nowadays, the observed and experienced risk factors are different from what scientific resources have suggested. In my opinion, the disease at the age of below 40 is more prevalent, which is on the contrary to what specialists previously believed. The number of these patients is relatively high. Still, the fact that the risk increases with age is undeniable". (P.25)

### Different perceptions and opinions

On the other hand, there were certain categories mentioned in each group that were not expressed by the other group.

#### Belief in supernatural powers

The majority of the lay participants believed that God’s will and destiny were involved in BC occurrence.

One of the participants whose brother and child had serious renal and mental problems stated:

"What God wants will come forward no matter what happens. We have three patients in our family. What about the end? He puts this disease in me. We have no choice, but to cope." (P.4)

Moreover, one of the participants with BC said:

"It was always in my mind that I would probably take my mother's cancer gene and I finally got it. They say whatever you think will happen. I always told myself that my mother was stressful and therefore, got cancer. . It was always in my mind that I will definitely get cancer. … .. we might have made some mistakes. God wants to test us this way. "(P.9)

#### Individual history of diseases

A history of disease was mentioned by the experts, which the lay participants were not aware of.

##### Previous breast problems

According to the experts’ interviews, factors like atypical proliferative hyperplasia, personal history of BC, and most importantly bilateral BC and history of breast biopsy are also among the risk factors of this disease. An expert mentioned:

"Other breast disorders are also known as risk factors. Breast disorders which are more proliferative than non-proliferative diseases, particularly atypical types of proliferative diseases. All these may be highly important risk factors, such as hyperplasia or atypical dysplasia..." (P.26)

##### Health problems

The experts explained that certain diseases may increase the risk of BC such as uterine fibroids, immunodeficiency status, cholecystitis, and infertility. They were mentioned as BC risk factors:

"We know that women with high estrogen are more likely to get uterine fibroids and cholecystitis. If patients do not have cholecystitis by the time they are diagnosed with BC, we expect them to develop cholecystitis in the future, which is highly prevalent in BC patients. However, it must be further investigated in the general population; otherwise, we cannot rely on it." (P.29)

Some lay participants pointed out that the risk factors they found in scientific resources and health centers were not in accordance with the current scientific findings. One of the participants said:

"My mother did all the screenings. She has six children and all of them were breastfed completely. Our mother has used very few contraceptive pills during her life ... About my mother's lifestyle, she uses olive oil for cooking. Her nutrition is completely healthy. She always eats wholegrain bread. My mother is an active person and goes walking regularly. Thus, we were surprised when she was diagnosed with BC. My aunt is a sedentary person who does not follow a healthy lifestyle. She uses unhealthy oils and fried foods, but it (BC) did not happen to her. My mother has lived in a small and low-populated city without air pollution. I do not know what could be the reason behind the disease in my mother. As far as we know, nobody in our family has had breast cancer."(P.20)

Most experts stated that the BC risk factors in Iran are different from those in Western countries. According to them:

"Factors such as alcohol and smoking are not very common in our patients. A large percentage of our patients do not have a specific and bold risk factor. Another issue is ageing, which is being discussed all over the world; with the increase in age, the risk of BC increases. We also generally accept age as a risk factor. However, because our overall population structure is relatively younger, we generally see patients at a younger age" (P.22)

Another point in the laywomen’s interviews was the source of information about the risk factors and symptoms of BC. The participants obtained their information from sources such as physicians or counselors, Internet, social networks like Instagram, peer groups, television programs, and, to a very small number, through books. At the same time, they stated that information about BC is less accessible, simple, and understandable for Iranian laywomen.

One of them stated:

"I think we all go to a gynecologist at least once every two or three years. I think it is highly effective if the doctor gives a brief explanation (about BC risk factors, signs and symptoms). I see a lot of women whose cancer progressed because they did not have proper understanding of the disease … There are very few television programs about BC. If sometimes there is a TV program, it is either too long or specialists use medical terms that we do not understand. I think it is much better to speak briefly and understandably … " (P.25)

## Discussion

This qualitative study aimed to explore the Iranian women’s perception of the risk factors of BC and compare them to the experts’ opinions. The key findings obtained from the data analysis indicated that the lay participants’ perception of risk factors for BC was incomplete and there was an obvious gap between the public versus experts’ perceptions. This is indicative of the need to improve their perception of BC risk factors. According to our results, the lay participants had lack of risk perception and awareness of some of the risk factors, such as reproductive history, exposure to ionizing waves, certain diseases, such as breast benign disease and some types of cancers, alcohol consumption, radiation exposure, in addition to anthropometric, and demographic characteristics. According to the results of the present study, both groups of the participants (laywomen and experts) believed that in numerous cases, the risk factors of BC are different from the scientific literature because based on their experiences, most women with BC have none of these risk factors. The experts stated that many Iranian women with BC do not have any of the hormonal, nutritional, or lifestyle-related risk factors. The laywomen perceived this issue as a contradiction between their experiences with the experts’ opinions. As another finding, some of the factors that the laywomen mentioned as a risk factor of BC were on the contrary to the opinions of the experts and scientific texts, such as supernatural powers as a risk factor for BC. Also, the views of experts in various fields on the risk factors for BC were often similar because their views are based on scientific evidence. The only difference was the effect of exposure to electromagnetic fields, and the experts who disagreed on this were both experienced oncologists.

Published scientific literature on the risk factors of this disease has highlighted the importance of modifiable lifestyle-associated behaviors in controlling and modifying cancer risk [27]. The World Health Organization has indicated that more than 30.0% of cancer mortalities could be prevented by modifying or avoiding behavioral or lifestyle-related risk factors [28].

Some of the risk factors which were not properly perceived are among the modifiable risk factors. Awareness of such risk factors could be conductive to the improvement in health promoting behaviors, increase in health literacy, and thus, perceived control over the BC. One’s perceived ability to control his or her health contributes to adopting healthy behaviors, such as lifestyle improvements and participation in screening programs [29].

Based on the results obtained herein, all the participants believed that lifestyle-related factors, such as sedentary life, smoking, lack of regular checkups, and unhealthy diet could increase the risk of BC. Meanwhile, there was lack of perception and awareness among the laywomen about the relationship between alcohol consumption and overweightness or obesity with BC whereas they are of the most consistently reported risk factors in the literature and experts’ opinions. Other qualitative studies on BC risk perception, in line with our results, have revealed that many of the participants considered alcohol irrelevant to BC and this relationship was largely unknown [9, 30]. Furthermore, in Iranian studies, the impact of increased body mass index on increasing the risk of BC has been confirmed [31].

The hormonal contraception methods, specifically OCPs, was identified as a risk factor of BC by all the participants. There was nevertheless a lack of perception and awareness about reproductive factors, including early age at menarche, late age at menopause, low parity or nulliparity, and no/low breastfeeding, as BC risk factors. Meanwhile, according to the experts’ opinions, endogenous reproductive risk factors can play a pivotal role in BC. In line with this result, in other studies in different countries, the factors linked to reproductive history, other than the use of contraception and hormones, were less frequently stated by the participants [9, 32].

According to the experts’ opinions, one of the risk factors of BC was exposure to radiation for medical purposes (diagnostic or therapeutic). None of the laywomen identified medical diagnostic role as a risk factor for BC. In this study, the laywomen were more likely to attribute BC to non-ionizing than to ionizing radiation; this may reveal a poor understanding of the difference between the types of radiation. Similar results have been obtained in other papers [8, 32, 33].

According to the experts’ opinions, positive history of breast benign disease, breast atypical hyperplasia, or some problems of other organs, such as uterine fibroids, infertility, cancers in other parts of the body, and immunodeficiency, could be the risk factors of BC. However, none of the participants considered the role of the aforementioned diseases as BC risk factors. This has been also reported in other studies [5, 34]. On the other hand, some expert opinions about the potential BC risk factors were not necessarily consistent with scientific literature, such as uterine fibroids as a risk factor. Herein, the experts had diverse opinions about some risk factors, such as stress, exposure to telecommunication noise, and excessive use of cosmetics. Since these factors remain controversial in the scientific evidence, the experts commented based on their personal experiences.

Superstitious beliefs exists throughout the world, which have been identified as a condition in which a person believes that certain actions can lead to certain consequences; this obviously has no scientific basis [35]. As a result, superstitious individuals may believe that diseases are controlled by unknown events and supernatural powers and therefore, find disease prevention strategies useless [36]; for instance, they assume that what you think about will happen or that showing happiness and joy in front of others will cause misery [8]. This kind of belief becomes destructive when it comes to health and affects people’s well-being since it becomes a part of the individual’s health beliefs [8, 35].

In the present study, the laywomen’s perception about BC risk factors comprised a mixture of cultural beliefs and the scientific knowledge disseminated by the media, internet, and health services. Previous research has reported similar findings that TV and radio programs and internet were identified as the main sources of information about BC among women and men [34, 37].

Generally, modifiable risk factors of BC, as reproductive factors, radiation exposure, intake of hormonal drugs, and lifestyle-related risk factors, were reported by experts, yet may be less well understood in the general population [38]. Increased laywomen’s perception and awareness of the modifiable risk factors of BC could increase the adherence to BC prevention strategies. Accordingly, improving public awareness and correcting women’s perception about BC risk factors and symptoms are believed to be of great necessity [39].

### Strengths and limitations

The current study has certain strengths; primarily it is the first study in Iran exploring Iranian women’s perception of BC risk factors through a qualitative method. Secondly, the comparison between the understandings of laywomen with the opinions of experts could to some extent determine the gap between scientific and lay perception of risk factors. On top of that, the use of participants with and without BC could have contributed to more accurate understanding of the public attitude towards this disease. This would also help specialists to improve public awareness in this regard.

As a limitation of this study, these results are valid within the Iranian or similar cultural contexts due to the limited generalizability of qualitative studies. Therefore, further qualitative investigation in this field could be recommended in communities with different cultures.

## Conclusion

In the present study, the lay participants’ perception about BC risk factors was found to comprise a mixture of cultural beliefs and the scientific knowledge dispersed by the media, internet, and health services. This study highlighted the need for health promotion and communication efforts in order to decrease the gap between lay and expert opinion on beliefs about the risk factors of BC. The highest proportion of the participants obtained their information from the television/radio for the first time. It seems necessary that mass media focus on refining general perception and increasing knowledge about BC in a transparent manner in their educational programs. In order to elevate awareness regarding BC, with focus on the role of screening and prevention, it is strongly suggested that a well-designed health education program be conducted to resolve the observed knowledge gaps. Programs that are accessible for women and consider their role in BC prevention should be included in the national strategies. For women to make risk-reducing changes in their lifestyle, they need to be educated about common BC risk factors.

The rich evidence generated by this qualitative research would assist clinicians regarding the range of variables that may affect the perception of risk factors by their clients. Moreover, it could be proposed that proper training and informative programs be considered when warning women about the risk of BC.

## Data Availability

All data generated or analyzed during this study are presented in this manuscript.

## Abbreviation

BC: Breast Cancer
